# Theoretical investigation of parallel ^63^NiO/GaP heterojunction nuclear battery with graphene layer and its time-related performance

**DOI:** 10.1038/s41598-025-91929-6

**Published:** 2025-03-04

**Authors:** Yang Zhao, Xinxu Yuan, Jingbin Lu, Xiaoyi Li, Renzhou Zheng, Qiming Cui, Yu Zhang, Haolin Li, Xinrui Liu, Ke Zhang, Haoran Gu, Hongyi Tian, Chunmiao Han, Lei Liang, Wei Chen, Yugang Zeng

**Affiliations:** 1 College of Physics, Jilin University, Changchun, 130012 China; 2 State Key Laboratory of Transient Optics and Photonics, Xi’an Institute of Optics and Precision Mechanics, Chinese Academy of Sciences, Xi’an, 710119 China; 3 School of Physics, Changchun University of Science and Technology, Changchun, 130022 China; 4 State Key Laboratory of Luminescence and Applications, Changchun Institute of Optics, Fine Mechanics and Physics, Chinese Academy of Sciences, Changchun, 130033 China; 5 Center of Materials Science and Optoelectronics Engineering, University of Chinese Academy of Sciences, Beijing, 100049 China; 6 Peng Cheng Laboratory, Shenzhen, 518000 China

**Keywords:** Nuclear physics, Nuclear energy, Materials for devices

## Abstract

Betavoltaic (BV) batteries are regarded as appealing power sources due to their high energy densities and long lifetimes. However, the low efficiency and maximum output power density of conventional BV batteries due to the self-absorption effect of radioactive sources, which consist of separate beta-radioactive sources and semiconductor absorbers, limit their applications. In this work, we optimized and compared six ^63^NiO-related heterojunction nuclear batteries utilizing Monte Carlo software Geant4 and finite element analysis software COMSOL Multiphysics. The ^63^NiO-related heterojunction nuclear batteries integrate beta-radioactive sources and semiconductor absorbers to overcome the shortcomings of conventional BV batteries. Furthermore, we proposed a parallel connection structure utilizing graphene electrode layer to connect two ^63^NiO/GaP heterojunctions based on the optimal one from the six heterojunctions in order to maximize the maximum output power density. The total energy conversion efficiency is 2.68% and the maximum output power density is $5236.2\hbox { nW}\cdot \hbox {cm}^{-2}$ of the parallel connection nuclear battery. Finally, we investigated the time-related performance of the parallel connection structure nuclear battery within 200 years. It shows that the maximum output power density decreases from $5236.2\hbox { nW}\cdot \hbox {cm}^{-2}$ in the beginning to $1330.5\hbox { nW}\cdot \hbox {cm}^{-2}$ at 200 years.

## Introduction

BV batteries are regarded as an alternative power source due to their high energy density, long lifetime and strong environmental adaptability^1,2^. Conventional planar BV batteries consist of a beta radioactive source and a semiconductor energy converter. The operational principle of BV batteries is that the beta particles emitted from radioactive sources interact with semiconductors and generate large quantities of electron–hole pairs (EHPs) via impact ionization, and then these EHPs are separated by the built-in electric field of the energy converter and collected by the electrodes to form the radiation-induced current.

For conventional BV batteries, the energy conversion efficiency and maximum output power density are low due to the self-absorption of radioactive sources^3–5^. The shortcomings limit the application of BV batteries. In the past few years, most of the research focused on optimizing the structure parameters of the energy converter to reduce energy loss, augment energy deposition and improve the collection efficiency to raise energy conversion efficiency and maximum output power density^6–11^. The structure parameters include the thickness of the beta radioactive source, the thicknesses of p-type semiconductor and n-type semiconductor, the donor concentration and acceptor concentration. Wide bandgap semiconductor nuclear batteries have obtained increasing attention due to their higher energy conversion efficiency and corresponding higher maximum output power density^11–13^. However, the energy conversion efficiency remains relatively low. This is because the beta-radioactive source and semiconductor converter of conventional nuclear batteries are separated, resulting in most of the energy of the beta radiation source being wasted inside the radiation source and failing to fundamentally solve the problem of low energy conversion efficiency. Therefore, only by solving the problem of self-absorption of the beta-radioactive source can we fundamentally resolve the low energy conversion efficiency of conventional nuclear batteries. In addition, due to the self-absorption effect of the beta-radioactive source, the maximum output power density of the nuclear battery will reach a saturation value and can no longer increase when the beta-radioactive source reaches a certain thickness^7^. Recently, some researchers have changed the distribution of radioactive source and energy transducer, or have combined radioactive source and electrode or have combined radioactive source and energy transducer. These modifications have significantly improved energy conversion efficiency and maximum output power density. McNamee et al. and Wagner et al. utilized nanowire structures to reduce the self-absorption energy loss^14,15^. Yakimov et al. utilized ^63^Ni as the electrode to reduce the energy loss in conventional metal electrode^16^. Wang et al. and Yuan et al. combined ^63^Ni with NiO to form ^63^NiO^17,18^. Among these studies, the last two achieved the best energy conversion efficiency. But which semiconductor material combined with ^63^NiO forming corresponding heterojunction can achieve the optimum conversion efficiency is still a question, and the minimum thickness of ^63^NiO corresponding to saturation maximum output power density of the heterojunction is also an unknown question.

In this study, we selected six kinds of common semiconductor materials which are able to form heterojunctions with ^63^NiO^19–24^. These materials are Si, InP, GaAs, $\hbox {Al}_{0.3}\hbox {Ga}_{0.7}$As, GaP and diamond and they are arranged in sequence of increasing bandgap. Compared to other semiconductor materials, these semiconductor materials have the following advantages respectively. Si is dominant in electronic devices due to its mature manufacturing technology and low cost. InP is known for its high radiation resistance. GaAs is characterized by a low noise figure and good temperature stability. $\hbox {Al}_{0.3}\hbox {Ga}_{0.7}$As has a wider bandgap and higher carrier mobility than GaAs, which is advantageous for enhancing the open-circuit voltage of nuclear batteries. GaP has a high melting point and thus has the potential for high-temperature applications. Diamond stands out with its ultra-wide bandgap, high thermal conductivity and high carrier mobility. The energy deposition of these heterojunctions is simulated utilizing Geant4 and the J–V and P–V characteristics are simulated by COMSOL Multiphysics. Those heterojunctions are simulated in different thicknesses of ^63^NiO and different doping concentrations of the six materials to obtain an optimum maximum output power density and then to find an optimal heterojunction. To further improve the maximum output power density, we use graphene as an electrode to combine the two ^63^NiO/GaP heterojunctions in parallel connection. This doubled the maximum output power density compared with the single ^63^NiO/GaP heterojunction. Finally, we simulated the time-related performance of the parallel connection heterojunction within 200 years and gave a fitting formula of the maximum output power density.

## Energy deposition and electron–hole pair generation

The Monte Carlo software Geant4 (version: 11.0.3, September 2022) is utilized to simulate the energy deposition distribution of beta particles emitted from ^63^NiO of the six heterojunctions. Geant4 is a toolkit to create simulations of the particles or radiation through matter. Applications build on Geant4 can simulate any setup or detector and radiation source, and record chosen output of physical quantities due to source particles and secondaries interacting with the material of the setup. It is used by a large number of studies in a variety of application areas, including high energy physics, nuclear physics, medical physics and astrophysics^25^. In order to ensure complete energy deposition, the thicknesses of the six n-type semiconductors are all set to 0.1 cm (a cross-sectional area of $1\times 1$ $\hbox {cm}^2$). The densities of ^63^NiO, Si, InP, GaAs, $\hbox {Al}_{0.3}\hbox {Ga}_{0.7}\hbox {As}$, GaP and diamond are 7.06, 2.33, 4.81, 5.32, 4.85, 4.14 and $3.52\, \hbox {g}\cdot \hbox {cm}^{-3}$ respectively. Three interaction models, including G4DecayPhysics, G4EmStandardPhysicsWVI and G4RadioactiveDecayPhysics, are set in the PhysicsList.cc files of the six heterojunctions.

**Fig. 1:**
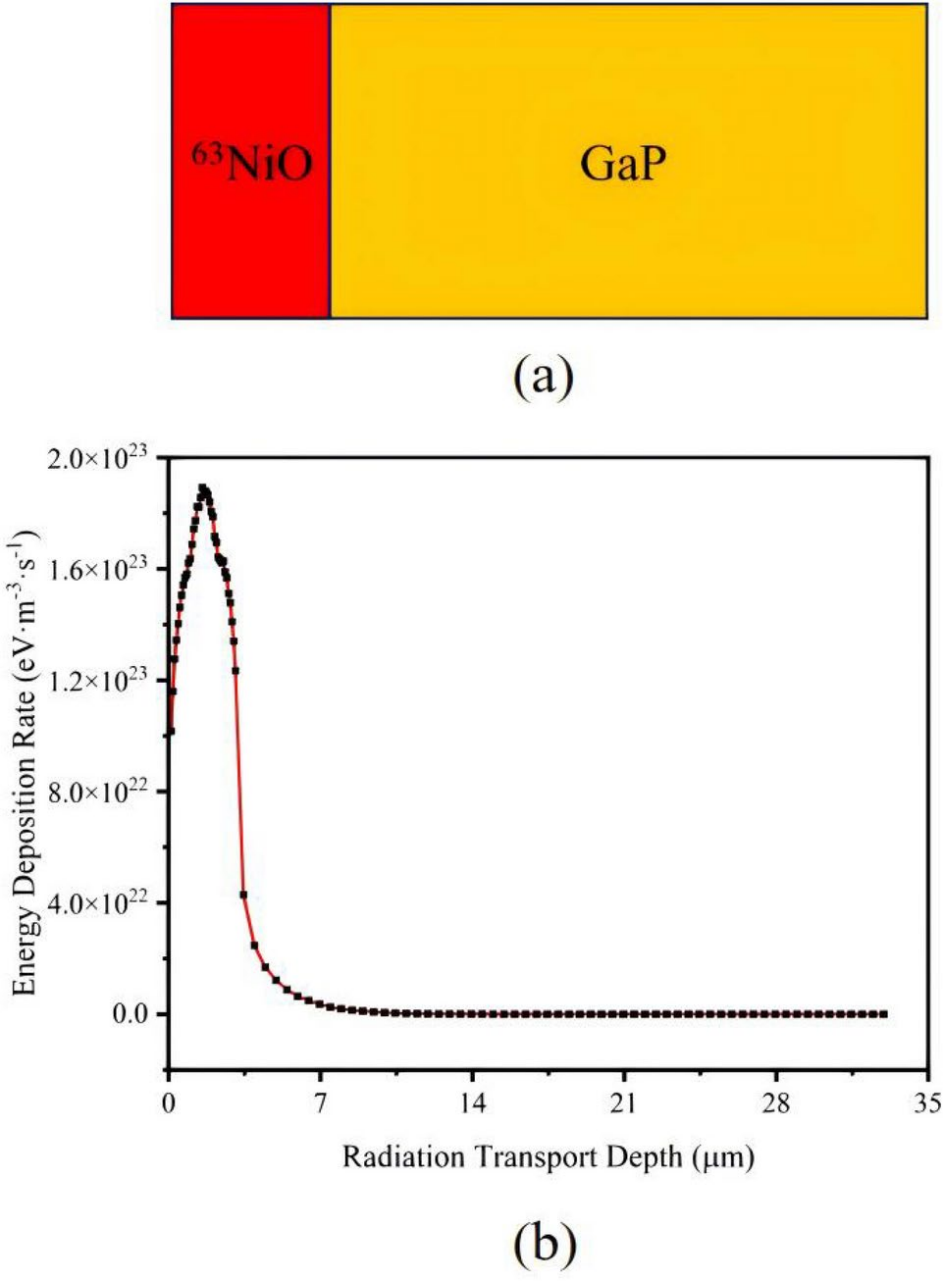
(**a**) Schematic diagram of ^63^NiO/GaP heterojunction. (**b**) Energy deposition rate vs radiation transport depth of ^63^NiO/GaP heterojunction. The thickness of ^63^NiO is 3.2 $\upmu \hbox {m}$.

The schematic diagram taking ^63^NiO/GaP heterojunction as an example is shown in Fig. 1a. Other heterojunctions have identical structures. The relationship between energy deposition in semiconductor materials and radiation transport depth taking ^63^NiO/GaP heterojunction as an example is shown in Fig. 1b. The thickness of ^63^NiO is $3.2\, \upmu \hbox {m}$. Other heterojunctions’ are similar. Furthermore, the relationship between electron-hole pair (EHP) generation rate [*G*(*x*)] and radiation transport depth is obtained due to the following formula^6^:

1 $$\begin{aligned} G(x)=\frac{E(x)}{E_{\text {ehp}}}, \end{aligned}$$

where *E*(*x*) is the energy deposition rate, $E_\text {ehp}$ is the mean ionizing energy. Herein, Bertuccio-Maiocchi-Barnett (BMB) relationship is utilized to calculate $E_\text {ehp}$ which is given by^26^

2 $$\begin{aligned} E_\text {ehp}=1.54 \ E_\text {g}+1.89 \, \text {eV}, \end{aligned}$$

where $E_\text {g}$ is the bandgap of the semiconductor material.

## Comparison and optimization

The finite element analysis software COSMSOL Multiphysics (version: 6.0, April 2022) is utilized to simulate the electrical characteristics of the heterojunction nuclear batteries. COMSOL Multiphysics is a powerful simulation software that integrates various physics and engineering disciplines, enabling users to model and analyze complex Multiphysics problems through finite element analysis. It is widely utilized in fields such as acoustics, electromagnetics, structural mechanics and semiconductor physics, allowing for detailed simulations that can predict system behavior under various conditions^27^. To make the simulation process efficient, the cross-sectional area of each heterojunction is set to $1\times 1 \upmu \hbox {m}^2$, and the thicknesses of the six n-type semiconductors are all set to $20\, \upmu \hbox {m}$. To explore the relation between the thickness of ^63^NiO and the kind of semiconductor material, the thicknesses of ^63^NiO are set as 0.05, 0.2, 0.8, 1.6 and $3.2\, \upmu \hbox {m}$ respectively among the above six heterojunctions. The minimum $0.05\, \upmu \hbox {m}$ is based on the preparation technique sol-gel spinning^28^. The x-coordinates of the left boundaries of ^63^NiO under the five thicknesses are all set to 0. The acceptor concentration of the p-type ^63^NiO is 10$^{16}$ $\hbox {cm}^{-3}$ and the donor concentration ($N_\text {d}$) of the five n-type semiconductors varies from $10^{13}$ to $10^{16}$ $\hbox {cm}^{-3}$ except that the donor concentration of n-type diamond varies from $10^{13}$ to $10^{15}$ $\hbox {cm}^{-3}$. These structure parameters will be optimized to maximize the maximum output power density.

The BV batteries are set as operating at room temperature (300 K), and several physical models are employed in the simulation. Firstly, the EHP generation rate is defined based on the Geant4 simulation results. Secondly, the analytic doping model is utilized to define the doping of p-type and n-type regions of the heterojunctions. Thirdly, the low-field mobility model is utilized to calculate the minority hole mobility ($\mu _\text {p}$),which is a function of $N_\text {d}$ and is given by^6^

3 $$\begin{aligned} \mu _\text {p}=\mu _\text {a}+\frac{\mu _\text {b}-\mu _\text {a}}{1+\left( \frac{N_\text {d}}{N_\text {ref}}\right) ^d}, \end{aligned}$$

where $\mu _\text {a}$, $\mu _\text {b}$, $N_\text {ref}$ and *d* are the fitting parameters^29–31^. In addition, the Shockley–Read–Hall (SRH) model is utilized to define the trap-assisted recombination. The minority hole lifetime ($\tau$) is also a function of $N_\text {d}$ and can be expressed as^6^

4 $$\begin{aligned} \tau _\text {p}=\frac{\tau _0}{1+\frac{N_\text {d}}{N_\text {R}}}, \end{aligned}$$

where $\tau _0$ is the intrinsic carrier lifetime and $N_\text {R}$ is the fitting parameter^32–38^. The semiconductor materials’ properties utilized in COMSOL Multiphysics are listed in Tables 1 and 2^39–41^.

**Table 1:** Properties of ^63^NiO utilized in COMSOL Multiphysics.

Property	Symbol	Value
Dielectric constant	$\varepsilon _\text {r}$	10.7
Bandgap (eV)	$E_\text {g}$	3.8
Electron affinity (eV)	$\chi$	1.46
Effective density of states in the conduction band ($\hbox {cm}^{-3}$)	$N_\text {c}$	$2.8\times 10^{19}$
Effective density of states in the valence band ($\hbox {cm}^{-3}$)	$N_\text {v}$	$1\times 10^{19}$
Minority electron mobility ($\hbox {cm}^2\cdot \hbox {V}^{-1}\cdot \hbox {s}^{-1}$)	$\mu _\text {n}$	12
Minority hole lifetime (s)	$\tau _0$	$1\times 10^{-6}$
Acceptor concentration ($\hbox {cm}^{-3}$)	$N_\text {a}$	$1\times 10^{16}$

**Table 2:** Properties of Si, InP, GaAs, $\hbox {Al}_{0.3}\hbox {Ga}_{0.7}$As, GaP and diamond utilized in COMSOL Multiphysics.

Symbol	Si	InP	GaAs	$\hbox {Al}_{0.3}\hbox {Ga}_{0.7}$As	GaP	Diamond
$\varepsilon _\text {r}$	11.7	12.5	12.9	12.05	11.1	5.7
$E_\text {g}$	1.12	1.344	1.424	1.798	2.26	5.5
$\chi$	4.05	4.38	4.07	3.74	3.8	0.35
$N_\text {c}$	$2.8\times 10^{19}$	$5.7\times 10^{17}$	$4.7\times 10^{17}$	$6.52\times 10^{17}$	$1.8\times 10^{19}$	$1\times 10^{20}$
$N_\text {v}$	$1.04\times 10^{19}$	$1.1\times 10^{19}$	$9.5\times 10^{18}$	$1.12\times 10^{19}$	$1.9\times 10^{19}$	$1\times 10^{19}$
$\mu _\text {a}$ ($\hbox {cm}^2\cdot \hbox {V}^{-1}\cdot \hbox {s}^{-1}$)	130	10	20	5	10	0
$\mu _\text {b}$ ($\hbox {cm}^2\cdot \hbox {V}^{-1}\cdot \hbox {s}^{-1}$)	500	170	491.5	240	147	2016
$N_\text {ref}$ ($\hbox {cm}^{-3}$)	$8\times 10^{17}$	$4.87\times 10^{17}$	$1.48\times 10^{17}$	$1\times 10^{17}$	$1\times 10^{18}$	$3.25\times 10^{17}$
*d*	1.25	0.62	0.38	0.324	0.85	0.73
$\tau _0$ (s)	$4\times 10^{-4}$	$8.5\times 10^{-8}$	$2\times 10^{-8}$	$3.15\times 10^{-8}$	$1\times 10^{-6}$	$2\times 10^{-6}$
$N_\text {R}$ ($\hbox {cm}^{-3}$)	$7.1\times 10^{15}$	$9.4\times 10^{17}$	$2\times 10^{18}$	$2\times 10^{18}$	$3.1\times 10^{14}$	$1\times 10^{15}$

**Fig. 2:**
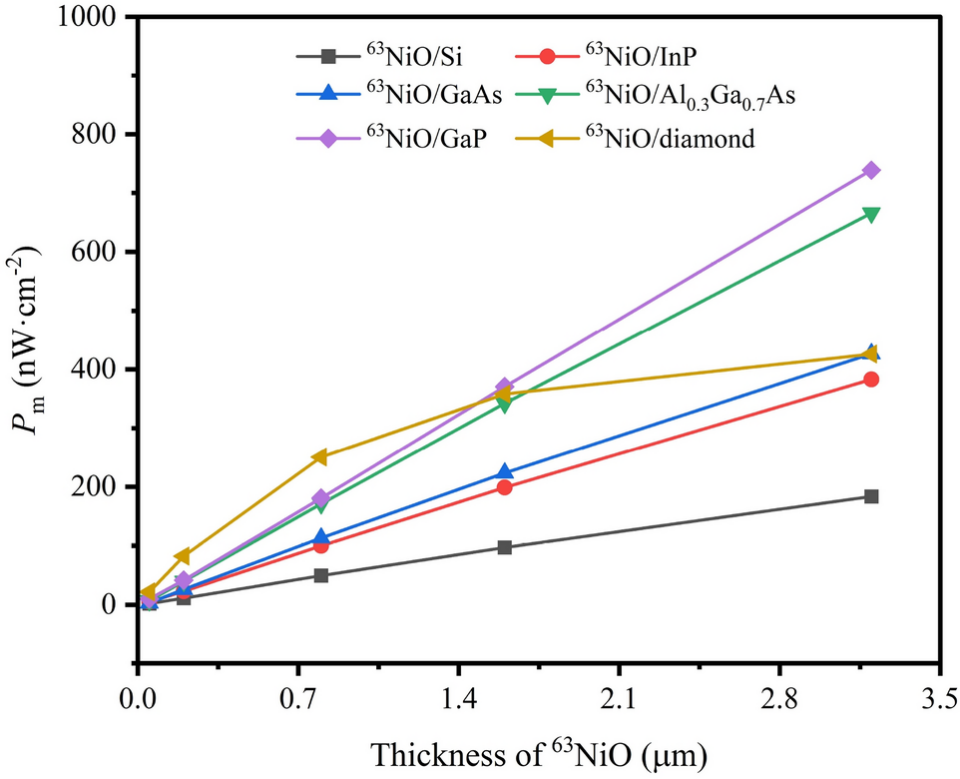
$P_\text {m}$ vs thickness of ^63^NiO for the six heterojunctions.

The simulation results show that for different heterojunctions, the optimized donor concentrations corresponding to maximum value of maximum output power density ($P_\text {m}$) are not totally identical. To be specific, the optimized concentrations are $10^{16}$, $10^{16}$, $10^{16}$, $10^{16}$, $10^{15}$ and $10^{13}$ $\hbox {cm}^{-3}$ for ^63^NiO/Si, ^63^NiO/InP, ^63^NiO/GaAs, ^63^NiO/$\hbox {Al}_{0.3}\hbox {Ga}_{0.7}$As, ^63^NiO/GaP and ^63^NiO/diamond heterojunctions respectively. The $P_\text {m}$ depending on the thicknesses of ^63^NiO for the heterojunctions is shown in Fig. 2. It shows that for different thicknesses of ^63^NiO, the optimal heterojunction is not always the same one based on $P_\text {m}$. To be specific, when the thickness of ^63^NiO is less than $1.6 \,\upmu \hbox {m}$, the optimal heterojunction is ^63^NiO/diamond. However, when the thickness of ^63^NiO is more than $1.6\, \upmu \hbox {m}$, the optimal heterojunction is ^63^NiO/GaP. It also indicates that the $P_\text {m}$ increases with increasing thickness of ^63^NiO.

**Fig. 3:**
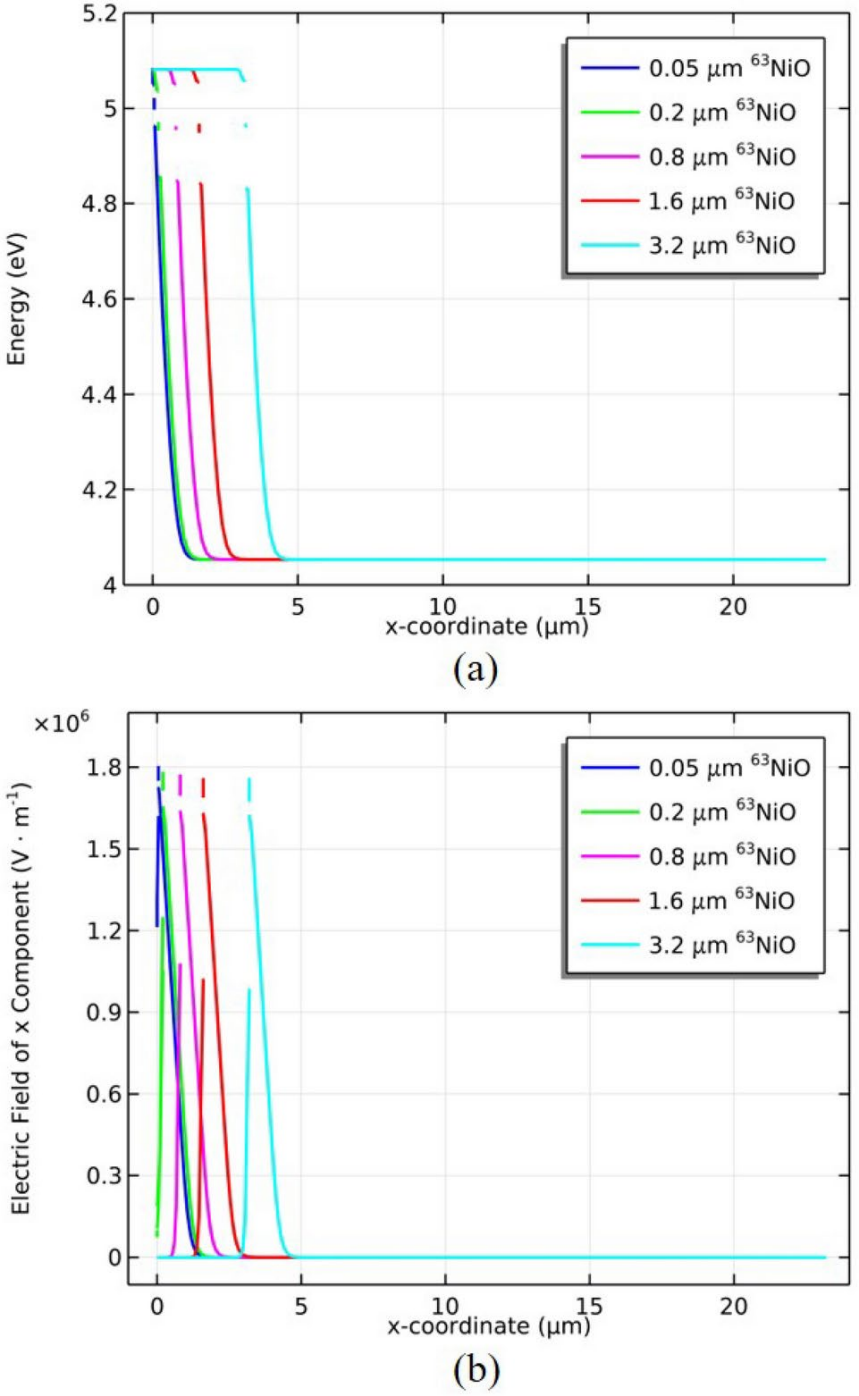
(**a**) Energy diagram of vacuum level of ^63^NiO/GaP heterojunction for the different thicknesses of ^63^NiO. (**b**) Electric field distribution of *x* component of ^63^NiO/GaP heterojunction for the different thicknesses of ^63^NiO.

Figure 3a shows the energy diagram of vacuum level of ^63^NiO/GaP heterojunction for the different thicknesses of ^63^NiO at thermodynamic equilibrium. In the depletion region, the vacuum level is bent and the depletion region width is $2.54\, \upmu \hbox {m}$. The built-in energy barrier of 1.03 eV is formed. The position of metallurgical junction moves right with increasing thickness of ^63^NiO. Figure 3b shows the electric field distribution of ^63^NiO/GaP heterojunction for the different thicknesses of ^63^NiO. The electric field is mainly distributed in the depletion region where the radiation-induced electron–hole pairs can be separated and it means that the electron–hole pairs generated in the depletion region can be collected more effectively.

**Fig. 4:**
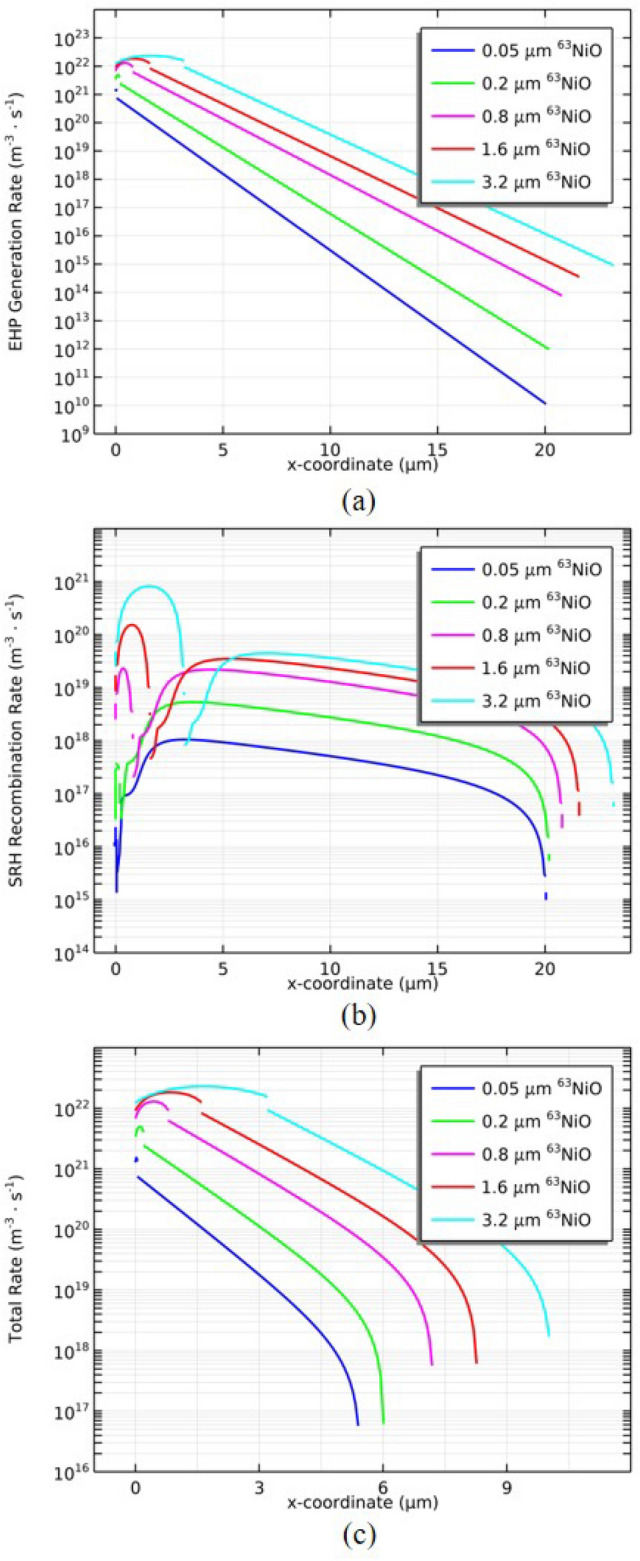
(**a**) EHP generation rate distribution of ^63^NiO/GaP heterojunction for the different thicknesses of ^63^NiO. (**b**) SRH rate distribution of ^63^NiO/GaP heterojunction for the different thicknesses of ^63^NiO. (**c**) Total rate distribution of ^63^NiO/GaP heterojunction for the different thicknesses of ^63^NiO.

Figure 4a shows the EHP generation rate distribution of ^63^NiO/GaP heterojunction for the different thicknesses of ^63^NiO. The EHP generation rate increases with increasing thickness of ^63^NiO due to the radioactivity increasing with increasing thickness of ^63^NiO. The radioactivity increasing leads to energy deposition increasing. In addition, the EHP generation rate increases first and then decreases in ^63^NiO and decreases exponentially in GaP for the same thickness of ^63^NiO. Figure 4b shows the SRH recombination rate distribution of ^63^NiO/GaP heterojunction for the different thicknesses of ^63^NiO. The SRH recombination rate of ^63^NiO is higher than GaP as a result of the higher EHP generation rate of ^63^NiO^11^. The SRH recombination is lower in the depletion region because the EHPs generated in it can be directly separated by the built-in electric field while the EHPs generated out of the depletion region diffuse into it and then be separated^17^. Figure 4c shows the total rate distribution of ^63^NiO/GaP heterojunction for the different thicknesses of ^63^NiO. The total rate is defined as EHP generation rate minus SRH recombination rate. The total rate increases with increasing thickness of ^63^NiO so that the short circuit current density ($J_\text {sc}$) will increase with increasing thickness of ^63^NiO.

**Fig. 5:**
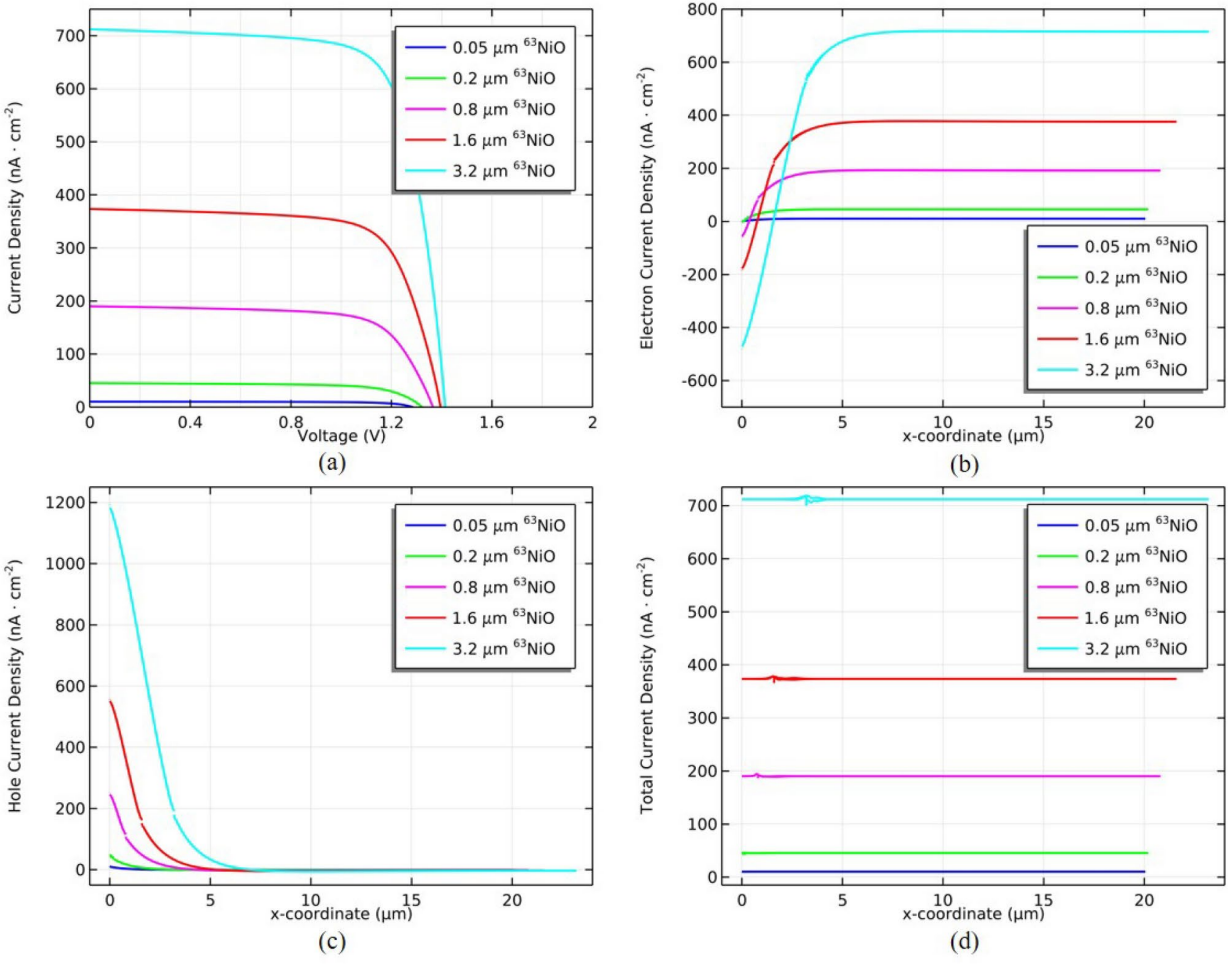
(**a**) J–V characteristics of ^63^NiO/GaP for the different thicknesses of ^63^NiO. (**b**) Electron current density distribution of 63NiO/GaP for the different thicknesses of ^63^NiO. (**c**) Hole current density distribution of ^63^NiO/GaP for the different thicknesses of ^63^NiO. (**d**) Total current density distribution of ^63^NiO/GaP for the different thicknesses of ^63^NiO.

Figure 5a shows the current density–voltage (J–V) characteristics of ^63^NiO/GaP heterojunction for the different thicknesses of ^63^NiO. It can be seen that the short current density increases with increasing thickness of ^63^NiO which can be attributed to the total rate increasing with increasing thickness of ^63^NiO. In addition, the open circuit voltage ($V_\text {oc}$) increases with increasing thickness of ^63^NiO which can be owing to the following formula^42^:

5 $$\begin{aligned} V_\text {oc}=\frac{kT}{q}\ln \left( {\frac{J_\text {sc}}{J_0}+1}\right) , \end{aligned}$$

where *k* is the Boltzmann constant, *T* is the temperature, *q* is the elementary charge and $J_0$ is the reverse saturation current density. It can be seen that the $V_\text {oc}$ increases with increasing $J_\text {sc}$. Figure 5b shows the electron current density distribution of ^63^NiO/GaP heterojunction for the different thicknesses of ^63^NiO. In the region of p-region left boundary, the diffusion current density is dominant compared with the drift current density for the minority carrier electron due to the higher EHP generation rate of the ^63^NiO part compared with the GaP part so that the electron density is negative. When it is close to the left boundary of the depletion region, the drift current density becomes dominant in comparison with the diffusion current density owing to the built-in electric field. Figure 5c shows the hole current density distribution of ^63^NiO/GaP heterojunction for the different thicknesses of ^63^NiO. In the p-region, the drift current density is dominant compared with the diffusion current density for the majority hole and the EHP generation is higher than n-region so that the hole current density is larger. Figure 5d shows the total current density distribution of ^63^NiO/GaP heterojunction for the different thicknesses of ^63^NiO. The total current density is electron current density plus hole current density. We can see that the total current density is consistent with the $J_\text {sc}$ in Fig. 5a.

**Fig. 6:**
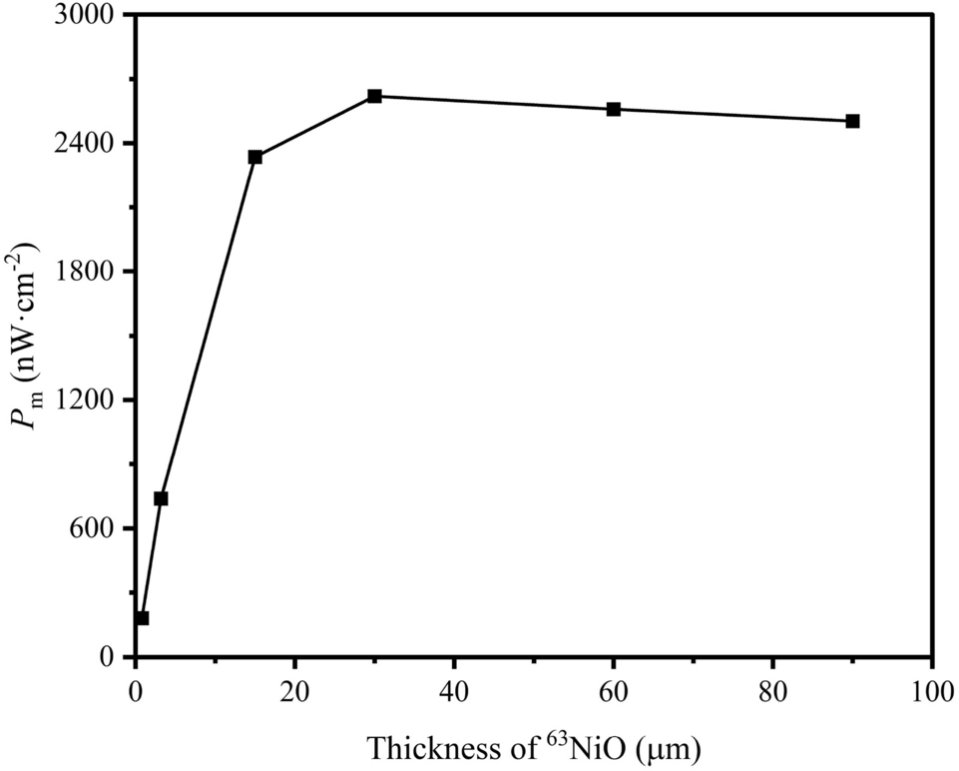
$P_\text {m}$ vs Thickness of ^63^NiO for ^63^NiO/GaP heterojunction.

We further studied the saturation $P_\text {m}$ based on ^63^NiO/GaP heterojunction. Figure 6 shows that $P_\text {m}$ arrives at the saturation value when the thickness of ^63^NiO is $30\, \upmu \hbox {m}$. With increasing thickness of ^63^NiO, the EHP pairs generated near the left boundary of ^63^NiO become more difficult to collect owing to recombination so that there will be a saturation value of $P_\text {m}$.

## Parallel connection structure

**Fig. 7:**
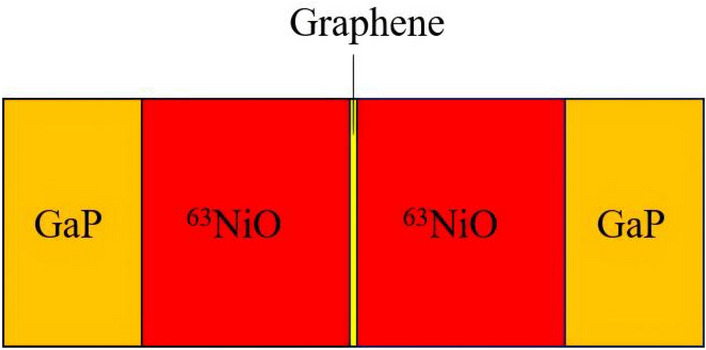
Schematic diagram of the parallel connection structure of double ^63^NiO/GaP heterojunctions with graphene.

In the above study, the converted energy is restricted by the single side of the beta particles emitted from ^63^NiO. To fully utilize the decay energy and further improve $P_\text {m}$, the two ^63^NiO/GaP heterojunctions were combined in parallel connection with graphene. Graphene has the advantages of large specific surface area, impressive electrical conductivity, high thermal conductivity, exceptional mechanical strength and excellent corrosion resistance. It has been used as an electrode material in electrochemical sensors, $\hbox {NO}_{2}$ gas sensors, supercapacitors, triboelectric nanogenerators^43–47^. Meanwhile, the atomic number of graphene is small so that it is able to minimally deposit the energy of beta particles when they pass through the middle layer compared with metal materials. As shown in Fig. 7, the thickness of ^63^NiO is $30\, \upmu \hbox {m}$, the thickness of GaP is $20\, \upmu \hbox {m}$ and the thickness of single layer graphene is 0.345 nm^48^. Taking the practical processing technique into consideration, we simulated the performance of the nuclear batteries with different layer numbers of graphene. The results are shown in Table 3. It indicates that the layer number has slight influence on the $P_\text {m}$ when the layer number is less than 10000. The comparison of the performance among the parallel connection ^63^NiO/GaP, the optimal single ^63^NiO/GaP, the conventional ^63^Ni-NiO/GaP and the conventional ^63^Ni-NiO/diamond heterojunction nuclear batteries is presented in Table 4. The thicknesses of ^63^Ni of the two conventional heterojunction nuclear batteries are both $5\, \upmu \hbox {m}$^7^ and the corresponding radioactivity is $9.918\times 10^{9}$ Bq.

**Table 3:** Simulation results of $J_\text {sc}$, $V_\text {oc}$ and $P_\text {m}$ with different layer numbers of graphene.

Layer number of graphene	$J_\text {sc}$ ($\hbox {nA}\cdot \hbox {cm}^{-2}$)	$V_\text {oc}$ (V)	$P_\text {m}$ ($\hbox {nW}\cdot \hbox {cm}^{-2}$)
1	4818.8	1.48	5236.2
10	4818.8	1.48	5235.2
100	4818.4	1.48	5231.2
1000	4813.6	1.48	5229.4
10000	4801.6	1.48	5217.4

**Table 4:** The comparison of the performance among the parallel connection ^63^NiO/GaP, the optimal single ^63^NiO/GaP, the conventional ^63^Ni-NiO/GaP and the conventional ^63^Ni-NiO/diamond heterojunction nuclear batteries.

Structure	$J_\text {sc}$ (nA$\cdot \hbox {cm}^{-2}$)	$V_\text {oc}$ (V)	$P_\text {m}$ (nW$\cdot \hbox {cm}^{-2}$)	*FF* $(\%)$	$\eta$ $(\%)$
Single ^63^NiO/GaP	2411.1	1.48	2618.3	73.37	2.68
Parallel ^63^NiO/GaP	4818.8	1.48	5236.2	73.42	2.68
^63^Ni-NiO/GaP	173.55	1.36	163.04	69.08	0.59
^63^Ni-NiO/diamond	93.691	3.89	341.85	93.80	1.23

## Time-related performance

**Fig. 8:**
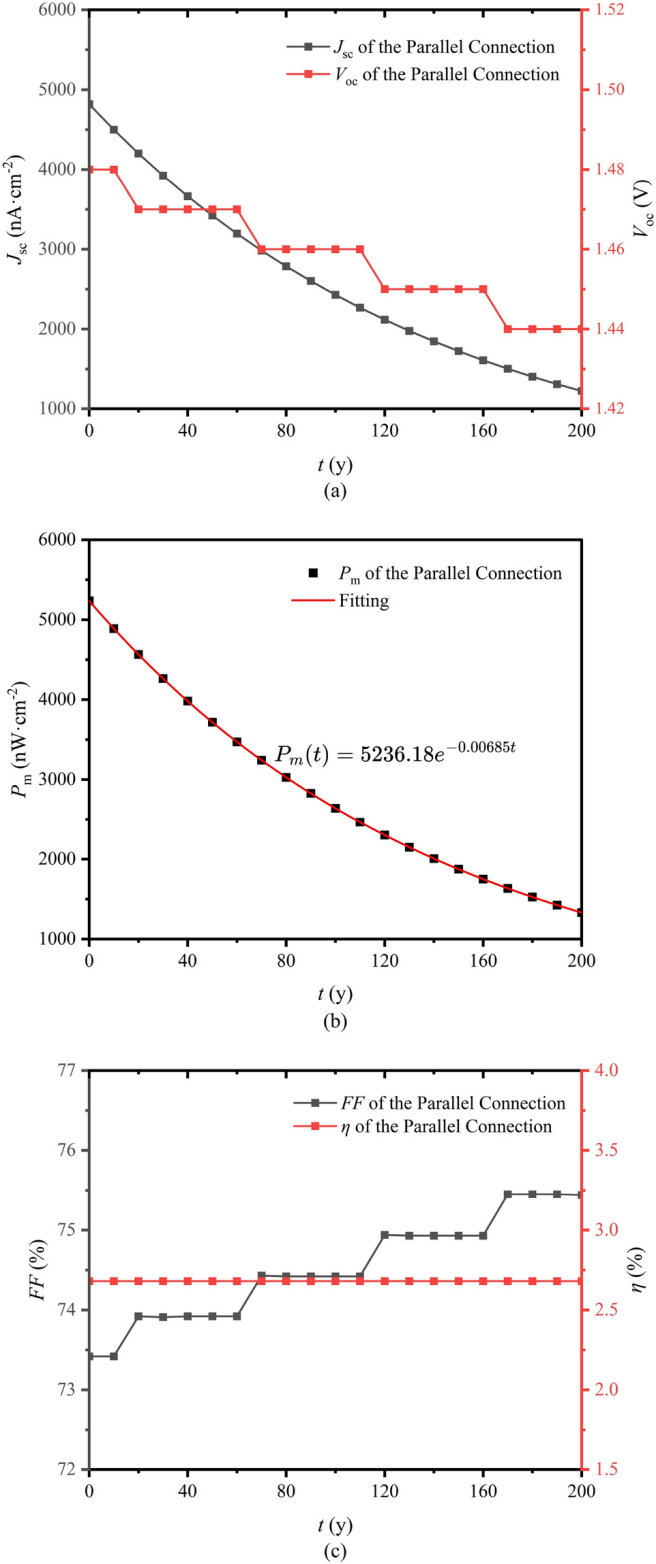
(**a**) Time-related $J_\text {sc}$ and $V_\text {oc}$. (**b**) Time-related $P_\text {m}$. (**c**) Time-related *FF* and $\eta$.

With time going by, the radioactivity of ^63^NiO decreases, and the electrical performance of the BV changes subsequently. The time-related performance of the parallel connection structure with single layer graphene is simulated. As shown in Fig. 8a,b, $J_\text {sc}$, $V_\text {oc}$ and $P_\text {m}$ decrease with increasing time because the deposited energy decreases with decreasing radioactivity EHP generation rate. As shown in Fig. 8c, *FF* increases with increasing time and $\eta$ remains constant. The *FF* is given by Ref.^49^

6 $$\begin{aligned} FF=\frac{P_\text {m}}{V_\text {oc}J_\text {sc}}\times 100\%, \end{aligned}$$

and the $\eta$ is given by^31^

7 $$\begin{aligned} \eta =\frac{P_\text {m}S}{1.602\times 10^{-19}AE_\text {ave}}\times 100\%, \end{aligned}$$

where *S* is the cross-sectional area ($S = 1 \hbox {cm}^2$), *A* is the radioactivity of ^63^NiO (the initial value of *A* is $7.001\times 10^{10}$ Bq) and $E_\text {ave}$ is the average energy of beta particles emitted from ^63^NiO ($E_\text {ave} = 17.425\hbox { keV}$).

In Fig. 8b, the formula of $P_\text {m}(t)$ is obtained through fitting, which can be utilized to predict the performance of the parallel connection BV battery within 200 years. We can see that the $P_\text {m}$ decreases from $5236.2 \hbox { nW}\cdot \hbox {cm}^{-2}$ at 0 y to 3717.6, 2639.4 and $1330.5 \hbox { nW}\cdot \hbox {cm}^{-2}$ at 50, 100 and 200 y respectively. Those value are 71%, 50% and 25% of the initial value. When the output power decreases significantly, the equipment may not be able to obtain enough power, resulting in unstable operation, or even malfunctions or shutdowns, affecting the normal function of the equipment and the completion of tasks. Since the decay of the radioactive source is not affected by the external environment, the decrease in activity caused by the decay of the radioactive source is inevitable, and the output power of the nuclear battery is inevitably reduced. Therefore, the potential mitigation strategy may only be to replace the nuclear battery with a new one.

## Conclusions

In this paper, to overcome the shortcomings of conventional planar BV batteries due to low efficiency and power density, a parallel connection structure of two ^63^NiO/GaP heterojunctions with a graphene layer was designed. The results exhibited a remarkable improvement of efficiency and power density. Herein, ^63^NiO/GaP heterojunction as one part of the parallel connection structure is based on the comparative research of six ^63^NiO-related heterojunctions. Through the Monte Carlo software Geant4 and the finite element analysis software COMSOL Multiphysics, ^63^NiO/Si, ^63^NiO/InP, ^63^NiO/GaAs, ^63^NiO/$\hbox {Al}_{0.3}\hbox {Ga}_{0.7}$As, ^63^NiO/GaP and ^63^NiO/diamond heterojunctions were investigated and compared on $P_\text {m}$. When the thickness of ^63^NiO is more than 1.6 $\upmu$m, the optimal heterojunction is ^63^NiO/GaP. It also indicates that the $P_\text {m}$ increases with increasing thickness of ^63^NiO, and the $P_\text {m}$ arrives at the saturation value when the thickness of ^63^NiO is $30\, \upmu \hbox {m}$. To further improve $P_\text {m}$, the two ^63^NiO/GaP heterojunctions were combined in parallel connection with graphene. In addition, the time-related performance was researched within 200 years based on the parallel connection nuclear battery. The $P_\text {m}$ decreases from $5236.2 \hbox { nW}\cdot \hbox {cm}^{-2}$ at 0 y to $1330.5\hbox { nW}\cdot \hbox {cm}^{-2}$ at 200 y. The final value is 25% of the initial value.

## Data Availability

The data that support the findings of this study are available from the corresponding author upon reasonable request.
